# A phase I/II trial of intraoperative breast radiotherapy in an Asian population: 10-year results with critical evaluation

**DOI:** 10.1093/jrr/rraa029

**Published:** 2020-06-04

**Authors:** Mariko Kawamura, Yoshiyuki Itoh, Takeshi Kamomae, Masataka Sawaki, Toyone Kikumori, Nobuyuki Tsunoda, Junji Ito, Yoshie Shimoyama, Hiroko Satake, Shinji Naganawa

**Affiliations:** 1 Department of Radiology, Nagoya University Graduate School of Medicine, Nagoya, Aichi, Japan; 2 Department of Breast Oncology, Aichi Cancer Center Hospital, Nagoya, Aichi, Japan; 3 Department of Breast and Endocrine Surgery, Nagoya University Graduate School of Medicine, Nagoya, Aichi, Japan; 4 Department of Pathology, Nagoya University Graduate School of Medicine, Nagoya, Aichi, Japan

**Keywords:** breast cancer, APBI, IORT, long follow-up, local control

## Abstract

Although phase III trials have been published comparing whole breast irradiation (WBI) with accelerated partial breast irradiation (APBI) using intraoperative radiotherapy (IORT), long-term follow-up results are lacking. We report the 10-year follow-up results of a prospective phase I/II clinical trial of IORT. The inclusion criteria were as follows: (i) tumor size <2.5 cm, (ii) desire for breast-conserving surgery, (iii) age >50 years, (iv) negative margins after resection and (v) sentinel lymph node-negative disease. A single dose of IORT (19–21 Gy) was delivered to the tumor bed in the operation room just after wide local excision of the primary breast cancer using a 6–12 MeV electron beam. Local recurrence was defined as recurrence or new disease within the treated breast and was evaluated annually using mammography and ultrasonography. A total of 32 patients were eligible for evaluation. The median patient age was 65 years and the median follow-up time was 10 years. Two patients experienced local recurrence just under the nipple, out of the irradiated field, after 8 years of follow-up. Three patients had contralateral breast cancer and one patient experienced bone metastasis after 10 years of follow-up. No patient experienced in-field recurrence nor breast cancer death. Eight patients had hypertrophic scarring at the last follow-up. There were no lung or heart adverse effects. This is the first report of 10-year follow-up results of IORT as APBI. The findings suggest that breast cancer with extended intraductal components should be treated with great caution.

## INTRODUCTION

Accelerated partial breast irradiation (APBI) can shorten the duration of treatment from 3–5 weeks to <1 week. The shortest APBI is intraoperative radiotherapy (IORT) which is performed during breast conserving surgery and means that the patient will not have to attend hospital after the surgery to receive adjuvant radiotherapy [1–5]. Based on multiple promising phase III results comparing whole breast irradiation (WBI) to APBI, the ASTRO guideline of APBI was updated in 2017 to relax the regulation [6, 7].

The two major phase III trials of IORT are the ELIOT trial [4] and the TARGIT trials [5]. In ELIOT, the 5-year local recurrent rates were significantly higher in IORT patients, at 4.4% for IORT and 0.4% for WBI, without significant difference in disease-free survival or overall survival. In the TARGIT A trial, the investigators reported that the 5-year local recurrent rates were significantly higher in the IORT group, at 3.3% for IORT and 1.3% for WBI, without significant difference in breast cancer death. However, in the TARGIT A trial, it was also reported that non-breast cancer deaths, specifically from cardiovascular causes and other cancers, were significantly higher in the WBI compared with the IORT group.

We also reported our 5-year follow-up results previously as a first in an Asian population, with no local recurrence in 32 patients and no Grade 3 (Common Terminology Criteria for Adverse Events version 3.0; CTCAE ver. 3.0) or greater adverse effects. However, 24% of patients experienced hypertrophic scarring, probably due to the large scar requirement for insertion of the shield disc, and no irradiation to the skin which is known to suppress the occurrence of hypertrophic scarring [8].

The major concern regarding the use of IORT as a method of APBI is that the follow-up time at publication has been too short, with the median follow up of 5 years for ELIOT and only ~2.5 years for TARGIT A. Considering that most patients included in those studies were at low risk for local recurrence, longer follow-up results are essential. Although this is not a phase III study, we believe that our 10-year follow-up results of IORT and a critical evaluation of the study is of interest for many radiation oncologists and other breast cancer specialists.

## MATERIALS AND METHODS

### Study design

The protocol for APBI using the IORT method has been described previously [8, 9]. The study protocol was approved by the institutional ethics committee and was registered with the University Hospital Medical Information Network (UMIN) clinical trial registry, number UMIN 000000018. Written informed consent was obtained from all patients prior to enrolment in the study. The inclusion criteria were as follows: (i) tumor size <2.5 cm, (ii) desire for breast-conserving surgery, (iii) age >50 years and (iv) negative margins after resection. In February 2009, the eligibility criteria were changed to include only patients with sentinel lymph node-negative disease, due to data supporting node-positive disease as a risk factor for local recurrence. For phase I of the study, the radiotherapy dose was escalated from 19 Gy/fraction to 21 Gy/fraction, incremented by 1 Gy per step. Each cohort comprised three patients and the recommended phase II dose was set at 21 Gy at 90% isodose.

### IORT procedure

After wide local excision of the primary breast cancer and sentinel node biopsy and/or axillary dissection, a single dose of IORT (19–21 Gy) was delivered via a Mobetron^®^ (Intraop Medical, USA) to the tumor bed using 6–12 MeV electron beams for phase I and 9 or 12 MeV for phase II patients (6 MeV was used for only the first three patients in phase I). The target area for radiation was at least 2 cm from the margins. Prior to irradiation, an acrylic resin-Cu disc with a diameter of 6–10 cm with 1-cm intervals was inserted between the mammary gland and the pectoralis muscle to shield the heart and lungs from unnecessary dosing [10]. The disc size was chosen to be larger than the applicator size. Patients were excluded from the study if their surgical margins were positive. Adjuvant chemotherapy and hormonal therapy were administered after the surgery if indicated.

### Study assessment

Acute and late adverse events were evaluated every 3 months for the first year after surgery and thereafter every 6 months for 5 years by breast surgeons and radiation oncologists independently based on CTCAE ver. 3.0. Local recurrence was defined as recurrence or new disease within the treated breast and was evaluated annually by mammography and ultrasonography. When local or distant recurrence was suspected, biopsy and magnetic resonance imaging (MRI) and/or computed tomography (CT) was performed.

### Statistical assessment

Local control rate was calculated using Kaplan–Meier statistics using EZR (Saitama Medical Center, Jichi Medical University, Saitama, Japan), which is a graphical user interface for R (The R Foundation for Statistical Computing, Vienna, Austria). More precisely, it is a modified version of R commander designed to add statistical functions frequently used in biostatistics [11].

## RESULTS

Between December 2007 and March 2010, 38 female breast cancer patients were recruited for the trial. Four patients were ineligible for IORT due to positive margins. One patient could not receive IORT due to technical problems relating to the radiation device. One patient received IORT but was excluded from the evaluation because the pathology indicated a metastatic tumor from primary lung cancer.

**Fig. 1.**
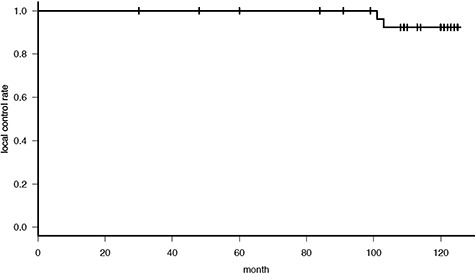
Local control rate.

**Fig. 2.**
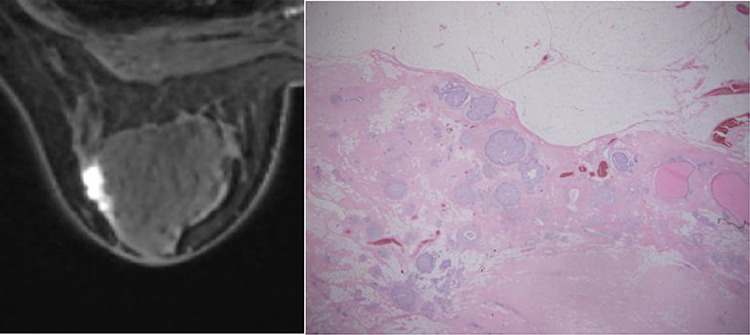
Dynamic contrast-enhanced MRI and pathological images of the primary tumor of patient #2 in Table 2 who had recurrence after 8 years follow-up. Axial view of delayed-phase (~5 min after gadolinium injection) T1-weighted image (left) shows linear enhancement toward nipple. She was diagnosed to have invasive cT1c tumor with extensive intraductal components. However, the resected specimen had intraductal components only (right).

**Fig. 3.**
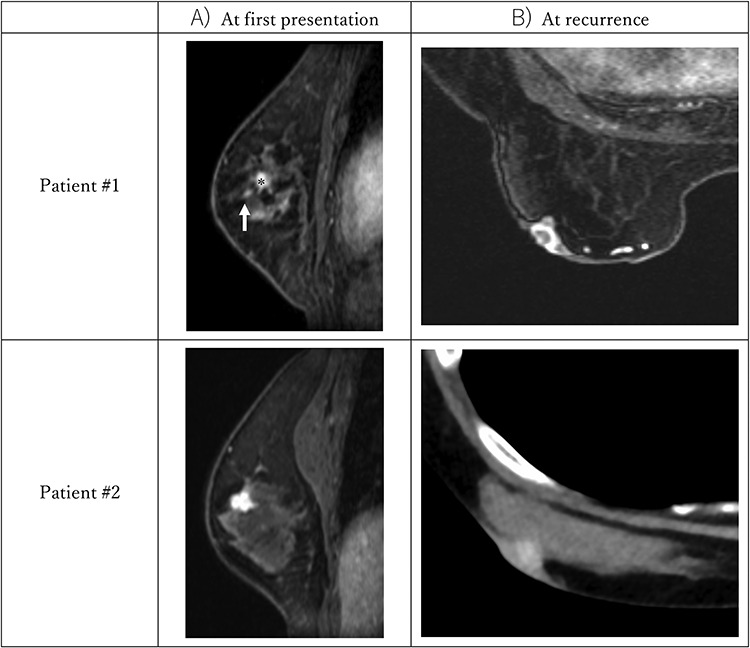
Dynamic contrast-enhanced MRI images of patients #1 and #2 (Table 2) at first presentation and MRI or CT image at recurrence. (**A**) Sagittal view of delayed-phase (~5 min after gadolinium injection) T1-weighted image (T1WI) of primary tumor at first presentation. Patient #1: a small enhanced nodule (↑) is captured in the nipple direction which indicates the EIC of the primary tumor (^*^). Patient #2: a 1.2 cm enhanced tumor is captured. In this view, the EIC is not completely clear, but as shown in Fig. 2, the axial view is suspected of having EIC. (**B**) Delayed-phase T1WI (achieved in the prone position) of patient #1 at recurrence and contrast enhanced CT image (achieved in the supine position) of patient #2 at recurrence. Enhanced tumor just under the nipple is captured in both patients.

A total of 32 patients were eligible for evaluation. The median patient age was 65 years (51–80 years) and the median study follow-up time was 10 years (2.5–10.5 years). Patient characteristics are described in Table 1. The 10-year local control rate was 92% as shown in Figure 1. Of 32 patients, one was lost to follow-up at 2.5 years and one was lost to follow-up after the protocol-specified 5 years of follow-up. Neither of these patients had experienced recurrence nor ≥Grade 3 adverse events at their last follow-up. Local ‘in-field’ recurrence was not observed in any patient; however, two patients experienced local recurrence just under the nipple after 8 years of follow-up. The characteristics of two local recurrent patients are as shown in Table 2. Patient #2 had 3 mm invasion in a biopsy specimen, thus with a preoperative MRI image, she was diagnosed to have cT1c invasive ductal carcinoma; however, the resected specimen had intraductal components only (Figure 2). Although they both had extensive intraductal components (EIC), both patients achieved a negative margin of >5 mm at primary surgery. One patient had nuclear grade 3 and one was HER2-positive but had not been treated with adjuvant trastuzumab. Both patients had hormone receptor-positive breast cancer and received hormonal therapy for 5 years after surgery. The primary tumor image and recurrent images are shown in Figure 3. The nipple was not irradiated in both cases. Both cases were operable at recurrence. One patient refused further treatment and further follow-up and the second patient received mastectomy and was in good health without re-recurrence at the time of writing.

**Table 1:** Patients characteristics (*n* = 32)

Characteristic		Number (rate, %)
Age, years	50–59	9 (28.1%)
	60–69	14 (43.8%)
	70–81	9 (28.1%)
Side	Left	14 (43.8%)
	Right	18 (56.2%)
Clinical T stage (preoperative)	Tis	3 (9.4%)
	T1a-b	9 (28.1%)
	T1c	20 (62.5%)
Pathological size, cm	Tis	3 (9.4%)
	<1	13 (40.6%)
	1–2.5	16 (50.0%)
Positive nodes	None	28 (87.5%)
	1	4 (12.5%)
Nuclear grades	G1, 2	28 (87.5%)
	G3	4 (12.5%)
Hormone receptor	ER^*^ and/or PgR^*^	29 (90.6%)
	ER− and PgR−	3 (9.4%)
HER2 status	Positive	3 (9.4%)
	Negative	29 (90.6%)
ASTRO consensus statement categories for the application of APBI	Suitable	25 (78.1%)
	Cautionary	3 (9.4%)
	Unsuitable	4 (12.5%)
Adjuvant systemic treatment	None	5 (15.6%)
	Hormonal therapy	22 (68.8%)
	Chemotherapy	3 (9.4%)
	Hormone and chemotherapy	2 (6.2%)

^*^ER estrogen receptor

^*^PgR progesterone receptor

Contralateral breast cancer was seen in three patients, one at treatment of the primary tumor, one at 3 years after surgery and one at 10 years after surgery, accounting for 9.4% of all patients. One patient experienced bone metastasis at 10 years and is currently taking hormonal therapy. Regional lymph node metastasis was not observed in any patient. There were no breast cancer-related deaths, but one patient died from gastric cancer after 4 years of follow-up. As reported previously [8], CTCAE Grade 2 fibrosis was experienced by three patients as an acute adverse event and by two patients as a late adverse event. There were no additional ≥Grade 2 late adverse events including hematoma, infection, pain or dermatitis. Hypertrophic scarring was observed in 10 patients 1 year after IORT, which decreased to seven patients in the 3-year follow-up without any treatment for hypertrophic scarring. One additional patient experienced hypertrophic scarring after 7 years follow-up at the surgical scar without any additional wound or trauma. One patient with hypertrophic scarring experienced progressive disease which required the application of external steroidal medication. However, this patient also experienced progressive hypertrophic scarring at the scar for sentinel lymph node biopsy (which was very small and distant from the IORT site). We therefore assume that this patient was at high risk for hypertrophic scarring regardless of the application of IORT. Other patients with hypertrophic scarring did not receive any treatment for hypertrophic scar. No patients experienced any lung or heart events during 10 years of follow-up.

**Table 2:** Characteristics of patients with ipsilateral recurrence

Characteristics	Patient #1	Patient #2
Age at first presentation, years	59	54
Side	Left	Right
Clinical size, cm	1.3	1.5
Pathological invasion size, cm	0.6	0.3
Positive nodes	None	None
Nuclear grade	G3	G1
Hormone receptor	ER^*^ and PgR^*^	ER^*^ and PgR^*^
HER2 status	Negative	Positive
ASTRO category	Suitable	Suitable
Adjuvant systemic treatment	Hormonal therapy	Hormonal therapy

^*^ER estrogen receptor

^*^PgR progesterone receptor

## DISCUSSION

Although two large phase III studies of APBI using the IORT technique have been published previously [3, 4], the results of more than 10 years follow-up have not previously been published. We previously reported that there were no ≥Grade 3 adverse events and, importantly, no additional adverse events from that point in this series in our 5-year follow-up. Two patients experienced local recurrence out of the irradiated field after they had completed their adjuvant hormonal therapy. Both patients had recurrence risks, one had nuclear grade 3 and the other was HER2-positive. They had been treated with hormone therapy for 5 years and the recurrence occurred 3 years after they stopped hormonal treatment. It is therefore apparent that longer hormonal treatment, such as for 10 years which is currently recommended, may have prevented this recurrence. Alternatively, a patient with HER2-positive disease may not have recurred if she had taken adjuvant trastuzumab as currently recommended. However, the fact that both cases recurred just under the nipple should be noted and so the patient with EIC should be treated with great deliberation.

The major disadvantage of IORT using electrons may be the fact that we cannot irradiate the nipple, thus a tumor with EIC may not be appropriately treated. Sawaki *et al.* reported the importance of the surgical procedure with IORT since skin is not irradiated with this technique. As a result, they experienced four cases of recurrence just under the skin near the primary tumor site [12]. We strongly agree with their report that surgical procedure as well as pathological evaluation of the resected species are very important factors to consider for optimal outcomes. Further, although routine use of enhanced MRI as preoperative diagnostic imaging is not strongly recommended, candidates for IORT are recommended to take enhanced MRI in order to detect as much EIC as possible.

The rationale for APBI is that >70% recurrence in ipsilateral breast occurs in the same quadrant as the primary tumor [13]. Thus, if we choose patients with a small risk of recurrence, the recurrence rate will be small enough to not to have statistical significance when compared with WBI. Further, if the irradiated field is smaller, the damage to the normal tissue, particularly lung and heart, will be smaller, and so the risk of non-breast cancer death may decrease. However, with 3D-imaging and use of the breath-hold technique [14], we can spare the heart and lung with WBI. Indeed, multiple studies are currently aiming to shorten the duration of WBI [15–17], and so the potential advantage of APBI is decreasing. However, with the IORT technique, adjuvant radiotherapy is completed during the operation and so any other APBI technique or WBI cannot approach the timeframe of IORT and we believe this is one of the major advantages of this approach.

Although we agree that IORT should be applied to low risk patients, considering the fact that lung and heart will not be irradiated with the IORT method [10], the main target of this treatment may be younger patients with very low risk for recurrence. After Darby *et al.* reported the adverse effect of irradiation dose to the heart in 2013 [18], many researchers have started to focus on adverse events from adjuvant radiotherapy, especially to the heart, as these typically manifest later. Jacobs *et al.* have also reported that patients <45 years old may experience higher myocardial infarction (MI) risk after left breast irradiation [19]. Young patients are at high risk for recurrence and so are thought to not be appropriate candidates for APBI. However, with breast cancer screening, there are many very small, low risk, but young patients. Lowering the risk of recurrence is important for young patients, but we should not expose them to higher risk for MI. There are many attempts to classify patients with a low risk of recurrence to avoid unnecessary irradiation. However, complete omission of irradiation may be difficult especially among young patients with very dense, rich breast tissue. IORT may therefore be an appropriate choice for those young, low-risk patients.

The 5-year local recurrent risk observed in PRIME II [20] is similar to that demonstrated in the ELIOT trial, which leads to the criticism that patients with very low risk for recurrence may be able to omit irradiation and may not even require APBI. However, it should be noted that patients included in ELIOT were younger than those included in PRIME II. Therefore, it is possible that some patients in ELIOT could have benefited from IORT. Further, in CALGB9343 [21], 10% of patients who had omitted radiotherapy experienced recurrence even though the investigators selected patients >70 years old. However, in contrast, only 2% of those who had been treated with radiotherapy in the 10 years follow-up experienced recurrence. Our finding is between these values which, although expected, should take into account the fact that 70% of our patients were <70 years old and patients who were >70 years old did not experience any recurrence.

As we have reported previously [8], one current major disadvantage of IORT is that hypertrophic scarring occurred more frequently than WBI and this may decrease cosmetic satisfaction. Knowing that darker skin populations (African, Spanish and Asian) tend to have higher risk of hypertrophic scarring [22], patients without risk of hypertrophic scarring should be chosen for IORT since the procedure leads to a larger scar and skin will not be irradiated. However, we believe that we could have treated those hypertrophic scars by surgery and low energy electrons if the patient had wished since the skin was not irradiated during the operation. Further, we are currently developing a new shield that can be inserted behind the tumor bed and that results in a smaller scar.

The major limitations of our study were that allocation to treatment was not randomized and that the number of patients included was small. However, if we can identify patients who are at low risk for recurrence in the young population, we can select them to receive IORT so that they will not have to undergo unnecessary irradiation to the heart or lung, and they do not also have to spend one more month receiving adjuvant radiotherapy. Further risk classification in young patients (i.e. <50 years old) is desirable as they could be the best candidates for IORT.

## CONCLUSION

This is the first report of 10 years of follow-up results of IORT as APBI. Local recurrence just under the nipple was seen in two patients, accounting for 6.2% of all patients after completion of hormonal therapy. This finding suggests that patients with EIC should be treated with great caution. IORT resulted in no additional heart and lung adverse events after the initial 5-years follow up and thus may be of benefit to certain patient subgroups. However, as reported previously, hypertrophic scarring occurs more frequently than WBI which may decrease cosmetic satisfaction.
